# Early evidence of SARS-CoV-2 in Milan, Jan-Feb 2020

**DOI:** 10.1186/s13052-021-01095-4

**Published:** 2021-06-30

**Authors:** Gregorio P. Milani, Giovanni Casazza, Antonio Corsello, Paola Marchisio, Alessia Rocchi, Giulia Colombo, Carlo Agostoni, Giorgio Costantino

**Affiliations:** 1 Pediatric Emergency Department, Fondazione IRCCS Ca’ Granda Ospedale Maggiore Policlinico, Milan, Italy; 2 Department of Clinical Science and Community Health, University of Milan, Milan, Italy; 3 Dipartimento di Scienze Biomediche e Cliniche “L. Sacco”, Università degli Studi di Milano, Milan, Italy; 4 Department of Pathophysiology and Transplantation, University of Milan, Milan, Italy; 5 Pediatric Highly Intensive Care Unit, Fondazione IRCCS Ca’ Granda Ospedale Maggiore Policlinico, Milan, Italy; 6 Pediatric Intermediate Care Unit, Fondazione IRCCS Ca’ Granda Ospedale Maggiore Policlinico, Via della Commenda 9, 20122 Milan, Italy; 7 Emergency Department, Fondazione IRCCS Ca’ Granda Ospedale Maggiore Policlinico, Milan, Italy

**Keywords:** SARS-CoV-2 infection, Children, Adults, Onset, Outbreak, Europe, Origin

## Abstract

**Background:**

A few studies have suggested that the Severe Acute Respiratory Syndrome coronavirus 2 (SARS-CoV-2) was present in Northern Italy several weeks before its official detection on February 21, 2020. On the other hand, no clinical data have been provided so far to support such hypothesis. We investigated clinical-epidemiological evidence of SARS-CoV-2 infection among children and adults referring to emergency department (ED) in the main hospital of the center of Milan (Italy) before February 21, 2020.

**Methods:**

A retrospective analysis of medical records of ED visits at the Fondazione Ca′ Granda Policlinico, Milan between January 11 and February 15 in 2017, 2018, 2019 and in 2020 was performed. The number of subjects referring with fever, cough or dyspnea was compared between the studied period of 2020 and the previous 3 years, by calculating a standardized referral ratio (SRR, number of observed cases in 2020 divided by the number of expected cases according to 2017–2019) and the corresponding 95% confidence interval (CI).

**Results:**

In the pediatric ED, 7709 (average 2570/year) and 2736 patients were visited during the period 2017–2019 and in the 2020, respectively. Among adults, 13,465 (average 4488/year) and 4787 were visited during the period 2017–2019 and in the 2020, respectively. The SRR was 1.16 (95% CI 1.10–1.23) in children and 1.25 (95% CI 1.16–1.35) in adults. The ratio for the two (children and adults) SRRs was 0.93 (0.84–1.02), suggesting a trend towards a higher frequency in adults compared to children.

**Conclusions:**

This study suggests that SARS-CoV-2 might have spread in Milan before February 21, 2020 with a minor trend among children.

## Background

In December 2019, a cluster of pneumonia of unknown etiology was reported in Wuhan, China [1]. Severe Acute Respiratory Syndrome coronavirus 2 (SARS-CoV-2) was later identified and its outbreak in Western Countries was first described in Lombardy (Italy) from February 21, 2020 [1]. However, based on serological studies, phylogenetic genomic analyses and environmental monitoring, SARS-CoV-2 might have been present in Northern Italy several weeks before [2–4]. Accordingly, an initial spread of the virus among young children was also hypothesized, given the seasonal incidence of viral outbreaks [5]. The question of diffusion among children is of interest to public health interventions, school attendance at first [6–8]. On the other hand, no supportive clinical-epidemiological data have been provided so far. To gauge the mentioned hypotheses, we investigated if there was any clinical-epidemiological evidence of SARS-CoV-2 infection among children and adults referring to emergency department (ED) in the main hospital of the center of Milan (the capital of Lombardy Region) before February 21, 2020. As secondary aim, we assessed if there was any difference between children and adults.

## Methods

A retrospective analysis of electronic medical records of the Fondazione Ca′ Granda Policlinico, Milan (Italy) registered between January 11 and February 15 in 2017, 2018, 2019 and in 2020 was performed.

Data on the reason for referral (main complaint) were retained and inserted in a predefined database. Then, the number of subjects referring with at least one of the symptoms consistent with SARS-CoV-2 infection (fever, cough or dyspnea) was compared between the studied period of 2020 and the previous 3 years. The three symptoms were chosen as they are the most frequent in both populations. Other complaints were not considered in this study since incidence and/or prevalence rates in children and adults may be very different thus affecting comparability [9]. Continuous and categorical data were presented as median and interquartile range or absolute frequency and percentage, respectively. Fisher’s exact test was used to compare the proportions of subjects referring with the symptoms of interest during 2020 with those observed in 2019, 2018 and 2017. In addition, an indirect standardization was performed, using the total number of cases in the period 2017–2019 as reference, by calculating a standardized referral ratio (SRR, number of observed cases in 2020 divided by the number of expected cases according to 2017–2019 data) with the corresponding 95% confidence interval (CI). The ratio between the adults and pediatric SRRs (with 95% CI) was calculated to compare the two populations. Finally, considering only patients presented to the EDs with symptoms consistent with SARS-CoV-2 infection, we compared the proportion of subjects admitted to the intensive care unit (ICU) in 2020 with those admitted to ICU in 2019, 2018 and 2017 (Table 1). *P* values lower than 0.05, two sided, were considered statistically significant.

**Table 1:** Characteristics of the Included Children and Adults (2017 to 2020, respectively) for the study period. Data of 2020 were compared with those obtained in 2019, 2018 and 2017

	Pediatric Emergency Department			
	2017	2018	2019	2020
**Total patients in ED,** N	2546	2444	2719	2736
**Age, years** median and IQR	2.5 [1.3–6.8]	3.1 [1.5–5.8]	2.4 [1.0–5.3]	3.4 [1.3–6.7]
**Females,** N (%)	1145 (45.0)	1126 (46.1)	1276 (46.9)	1176 (43.0)
**Reason for referral,** N (%)				
Dyspnea	108 (4.2)	122 (5.0)	115 (4.2)	98 (3.6)
Cough	241 (9.5)	200 (8.2)	249 (9.2)	224 (8.2)
Fever	527 (20.7)^*^	707 (28.9)^*^	743 (27.3)^*^	918 (33.6)
At least one of the previous	876 (34.4)^*^	1029 (42.1)^*^	1107 (40.7)^*^	1240 (45.3)
ICU admission, N (%)^a^	4/876 (0.5)	5/1029 (0.5)	3/1107 (0.3)	4/1240 (0.3)
	**Adult Emergency Department**			
**Total patients in ED,** N	4497	4422	4546	4787
**Age, years** median and IQR	52 [34–73]	53 [35–74]	53 [34–74]	51 [32–73]
**Females,** N (%)	2219 (49.3)	2163 (48.9)	2161 (47.5)	2349 (49.1)
**Reason for referral,** N (%)				
Dyspnea	233 (5.2)^*^	231 (5.2)^*^	307 (6.8)	302 (6.3)
Cough	56 (1.2)^*^	77 (1.7)	77 (1.7)	100 (2.1)
Fever	140 (3.1)^*^	138 (3.1)^*^	186 (4.1)^*^	240 (5.0)
At least one of the previous	429 (9.5)^*^	446 (10.1)^*^	570 (12.5)	642 (13.4)
ICU admission, N (%)^a^	1/429 (0.2)	1/446 (0.2)	0/570 (0)^*^	8/642 (1.2)

Comparisons with 2020: ^*^higher in 2020, *P* < 0.05; (two-sided Fisher’s exact test). ^a^the number of subjects with at least one symptom among dyspnea, cough or fever, is used as denominator

*Abbreviations*: *IQR* Interquartile Range, *ED* Emergency Department, *ICU* Intensive Care Unit

The Milano Area 2 ethics committee approved the study, which included a waiver of informed consent for participation, being the investigation retrospective.

## Results

A total of 7709 (average 2570/year) and 2736 patients were seen in pediatric ED during the period between 2017 and 2019 and in the 2020, respectively. In adult ED, a total of 13,465 (average 4488/year) and 4787 were seen during the period between 2017 and 2019 and in the 2020, respectively. A slight increase in the number of visits were observed for the pediatric (+ 6.4%) and adult (+ 6.7%) ED during 2020 as compared with the period 2017–2019. Table 1 shows the characteristics of patients during the considered periods. SRR was 1.16 (95% CI 1.10–1.23) in children and 1.25 (95% CI 1.16–1.35) in adults. The ratio for the two (children and adults) SRRs was 0.93 (0.84–1.02), suggesting that pediatric ED visits for the symptoms consistent with SARS-CoV-2 infection showed a trend towards a minor frequency compared to adults (despite not statistically significant). A similar trend was observed by comparing transfers from ED to ICU of either the pediatric or the adult ED in 2020, vs those extrapolated from the three previous years, respectively.

## Discussion

This analysis found an increased number of patients visiting the ED with fever, cough or dyspnea between January 11 and February 15, 2020 as compared to the three previous years. These results are consistent with the hypothesis of an increased number of ED visits possibly attributable to the presence of SARS-CoV-2 in Milan, the main center of Lombardy Region (Italy), before the first diagnosed case. On the other hand, children accessing ED with suggestive symptoms of such infection were not more frequent than adults.

Anecdotal reports have suggested that SARS-CoV-2 was already present among European adults in January 2020 [10]. Furthermore, two different studies conducted in Lombardy identified antibodies against the SARS-CoV-2 in sera of adult subjects, which were collected before February 20, 2020 [4, 11]. Among children, the SARS-CoV-2 RNA was detected in an oropharyngeal swab collected in December 2019 from a child living in Milan [12]. However, a study including more than 200 children with bronchiolitis and 49 healthy children enrolled between November 2019 and February 2020 failed to identify the SARS-CoV-2 RNA in the nasal swab of these subjects [13]. Taken together, these previously published data are in line with the findings of this retrospective clinical-epidemiological study, suggesting a possible early spread of SARS-CoV-2 infection mainly in the adult population. Since symptoms in children were at least not more frequent than in adults, a major role of children in disseminating symptomatic SARS-CoV-2 infections before the end of February 2020 is not supported by our observation.

This study has limitations. It is a retrospective monocenter study and no etiologic investigation has been performed, making the interpretation of the findings speculative. Furthermore, since only symptoms consistent with SARS-CoV-2 infection were investigated, the hypothesis that children are more infected but less symptomatic than adults cannot be dismissed. Yet, a previous report of our group conducted during the first lockdown in the same EDs has shown a SARS-CoV-2 infection prevalence of 1.2% in asymptomatic children and of 9.2% in a comparable adult population [14]. A similar study conducted during the school re-opening in the same setting was not able to find a higher prevalence of asymptomatic carriers among children as compared to adults [15]. Finally, it has been reported that children infected with SARS-CoV-2 might present several clinical presentations and therefore some potential cases could have been excluded in our analysis [16]. On the other hand, the symptoms considered as suggestive of a SARS-CoV-2 infection in this study are the most frequently reported both in symptomatic children and adults infected by the SARS-CoV-2 [17, 18]. A strength of this study is that data of 2020 were compared with those of the three previous years. This rather extended period of comparison is likely to reduce the impact of yearly variability of viral epidemics [19]. Moreover, safety protection devices (e.g., face masks), which can limit the spread of respiratory infections, were not yet systematically adopted neither in early 2020 nor in the previous years, thus reinforcing the comparability of the data.

## Conclusions

This monocenter study points out an increased number of patients visiting the ED with symptoms possibly consistent with a SARS-CoV-2 infection between January 11 and February 15, 2020 as compared to the three previous years, with a minor trend among children. These clinical-epidemiological data may further support the hypotheses of a spread of SARS-CoV-2 among the adult population in Italy before its official detection.

## Data Availability

Data are available at the corresponding author upon reasonable request.

## Abbreviations

SARS-CoV-2: Severe Acute Respiratory Syndrome coronavirus 2
ED: Emergency department
SRR: Standardized referral ratio
CI: Confidence interval
ICU: Intensive Care Unit
